# Genome-wide association study reveals a locus for nasal carriage of *Staphylococcus aureus* in Danish crossbred pigs

**DOI:** 10.1186/s12917-015-0599-y

**Published:** 2015-11-26

**Authors:** Per Skallerup, Carmen Espinosa-Gongora, Claus B. Jørgensen, Luca Guardabassi, Merete Fredholm

**Affiliations:** Department of Veterinary Clinical and Animal Sciences, University of Copenhagen, Frederiksberg, Denmark; Department of Veterinary Disease Biology, University of Copenhagen, Frederiksberg, Denmark

**Keywords:** Pigs, Staphylococcus aureus colonization, MRSA control

## Abstract

**Background:**

*Staphylococcus aureus* is an important human opportunistic pathogen residing on skin and mucosae of healthy people. Pigs have been identified as a source of human colonization and infection with methicillin-resistant *Staphylococcus aureus* (MRSA) and novel measures are needed to control zoonotic transmission. A recent longitudinal study indicated that a minority of pigs characterized by high nasal load and stable carriage may be responsible for the maintenance of *S. aureus* within farms. The primary objective of the present study was to detect genetic loci associated with nasal carriage of *S. aureus* in Danish crossbred pigs (Danish Landrace/Yorkshire/Duroc).

**Results:**

Fifty-six persistent carriers and 65 non-carriers selected from 15 farms surveyed in the previous longitudinal study were genotyped using Illumina’s Porcine SNP60 beadchip. In addition, *spa* typing was performed on 126 *S. aureus* isolates from 37 pigs to investigate possible relationships between host and *S. aureus* genotypes. A single SNP (MARC0099960) on chromosome 12 was found to be associated with nasal carriage of *S. aureus* at a genome-wide level after permutation testing (*p* = 0.0497) whereas the association of a neighboring SNP was found to be borderline (*p* = 0.114). Typing of *S. aureus* isolates led to detection of 11 *spa* types belonging to the three main *S. aureus* clonal complexes (CC) previously described in pigs (CC9, CC30 and CC398). Individual carriers often harbored multiple *S. aureus* genotypes and the host-pathogen interaction seems to be independent of *S. aureus* genotype.

**Conclusion:**

Our results suggest it may be possible to select pigs genetically resistant to *S. aureus* nasal colonization as a tool to control transmission of livestock-associated MRSA to humans.

**Electronic supplementary material:**

The online version of this article (doi:10.1186/s12917-015-0599-y) contains supplementary material, which is available to authorized users.

## Background

*Staphylococcus aureus* is a significant human pathogen causing wound and skin infections, endocarditis and bacteremia [1, 2]. It has long been recognized that healthy individuals may be colonized on skin and mucosae, the most frequent carriage site in humans being the anterior nares [3, 4]. Longitudinal studies have demonstrated the existence of three *S. aureus* nasal carriage patterns, i.e., persistent carriers (~20 % of the population), non-carriers (~50 %) and intermittent carriers (~30 %) [3, 4]. There is evidence showing that nasal carriage is associated with a higher risk of *S. aureus* infection [5, 6]. Colonization of the host is a complex process which is influenced by host factors, bacterial factors, and environmental factors [6–8]. Although the heritability of *S. aureus* carriage was not significant in two human studies [9, 10], single nucleotide polymorphisms (SNPs) associated with nasal carriage have been found for several candidate genes, e.g., C-reactive protein (*CRP*), interleukin 4 (*IL-4*), and glucocorticoid receptor (*NR3C1*) [11–15]. In addition, genetic loci associated with susceptibility to S. aureus infection have been reported in murine studies [16, 17]. Collectively, these studies suggest that host gene variants underlie differences in susceptibility to *S. aureus* colonization. However, candidate gene studies suffer from the limitation that they only assess the effects of specific genes picked by the researcher based on hypothesized involvement in disease etiology. In contrast, genome-wide association studies (GWAS) survey the entire genome, and many of the associations found in GWAS identify novel candidate genes [18, 19].

Pigs and other livestock species carrying methicillin-resistant *S. aureus* (MRSA) clonal complex (CC) 398 may act as a source of human colonization and infection [20, 21]. Spread of this livestock-associated MRSA clone is presently regarded as a threat to public health, and effective control measures preventing transmission of MRSA to farmers and other people exposed to livestock are urgently needed [22, 23]. A recent longitudinal study revealed that a minority of pigs characterized by high nasal load and stable carriage may be responsible for the maintenance of *S. aureus* within farms [24]. The objective of the present study was to detect loci associated with nasal carriage of *S. aureus.* Danish crossbreds classified as persistent carriers (*n* = 56) and non-carriers (*n* = 65) were genotyped by GWAS using Illumina’s Porcine SNP60 beadchip [25]. A single SNP on chromosome 12 was found to be genome-wide significant after permutation testing. The region of interest was inspected and we identified four candidate genes which may control *S. aureus* colonization in pigs.

## Methods

### Phenotypic characterization of pigs

Our study population comprised 56 persistent carriers and 65 non-carriers from 15 farms located in the central part of Jutland, Denmark (three specific-pathogen-free (SPF) and 12 non-SPF farms). Most pigs were phenotyped in the previous longitudinal study [24] and 21 additional pigs were recruited for this study on four of the farms surveyed in the longitudinal study. Nasal swabs (Dryswab™, MWE, UK) were collected from all pigs three times on a weekly basis. Pigs that were *S. aureus*-positive on all three sampling points were classified as persistent carriers whereas non-carriers were negative on at least two sampling points and with no more than 100 CFU/swab in the remaining sample. In order to ensure exposure to a minimum colonization pressure, non-carriers were included only if they originated from farms where at least one persistent carrier was detected. The distribution of persistent carriers and non-carriers among farms is shown in Additional file 1: Table S1. All pigs were crossbreeds (Danish Landrace/Yorkshire/Duroc) of approximately 70 kg. Pedigree details were not available but since the farmers used mixed semen to produce the offspring, the sample was expected to comprise a mixture of half-sibs (by sow and boar) and more distantly related pigs.

According to Danish laws (Danish Animal Experimentation Act, Chapter 1, Paragraph 1, point 3) no ethical approval was required for this study since the blood samples collected from the animals were taken for diagnostic purposes. All procedures concerning the animals were part of routine examinations and diagnosis of animals normally used at production farms. All handling of animals was performed by trained personnel and veterinarians.

### Genotyping of pigs

To detect QTLs associated with *S. aureus* carrier status, we genotyped all pigs using diagnostic blood samples collected in EDTA tubes (VWR, USA). DNA was extracted using either a salting out procedure with minor modifications [26] or MasterPure™ Complete DNA and RNA Purification Kit (Epicentre Biotechnologies, USA) according to the manufacturer’s instructions. The concentration and purity of DNA was measured on a NanoDrop 1000 spectrophotometer (Thermo Fisher Scientific, USA). 2500 ng of each sample was submitted for genotyping to GeneSeek, Inc. (http://www.neogeneurope.com). Samples were genotyped for 61,565 SNPs using Illumina’s Porcine SNP60 beadchip [25].

### Isolation and genotyping of *S. aureus*

To study possible relationships between host and *S. aureus* genotypes*, S. aureus* was isolated from 37 persistent carriers whose nasal swabs had been stored at−80 °C in the previous study [24]. Swabs were directly plated onto SaSelect agar (Biorad, USA) and enriched in Müller-Hinton broth containing 2.5 % of NaCl to enhance *S. aureus* detection. After overnight incubation one presumptive *S. aureus* colony was randomly selected for each sampling point. Additional colonies were isolated if they had clear morphological features suggesting the presence of different strains on the same plate. If *S. aureus* was not detected by direct plating, the enrichments were further processed as described above. All isolates were characterized by *spa* typing [27, 28] and *spa* types were assigned using Ridom Staphtype software, version 2.2.1 (Ridom GmbH, Würzburg, Germany). Associations between *spa* types and clonal complexes (CC) were determined according to information available in the scientific literature. For *spa* types not previously associated to a clonal complex, BURP cluster analysis (Ridom StaphType software, version 2.2.1) was used to infer association [27].

### Statistical analyses

Data were analyzed in R version 3.1.0 [29]. Genotype data were analyzed and visualized using the GenABEL package [30, 31] except for Manhattan plots which were made using the qqman package [32]. SNPs were excluded prior to analysis if genome position was not provided (*n* = 12,627) or if they were located on sex chromosomes (*n* = 1381). SNP genotype data were subjected to quality control (QC) measures. GenABEL applies QC filters using an iterative process; for individuals we used the following criteria, call rates > 0.95, false discovery rate (FDR) for unacceptably high heterozygosity < 0.01 and identity-by-state (IBS) < 0.95 (based on 2000 markers); for SNPs we used the following criteria (number of SNPs that did not pass the threshold), call rate > 0.95 (2500), minor allele frequency (MAF) > 0.05 (7012), and SNPs in Hardy-Weinberg equilibrium with *p*-values > 0.05 (15,073). After QC a total of 23,919 autosomal SNPs mapped to build Sscrofa 10.2 and 121 individuals (56 carriers, 65 non-carriers) were included in the final analysis.

We estimated the average relatedness between pigs by computing an *n* × *n* marker-based genomic kinship matrix for all pairs of pigs. Kinship coefficients between two individuals (average *identical-by-state* value) were estimated using 23,919 autosomal SNPs which had passed QC as described elsewhere [31]. The genomic kinship matrix was transformed to a distance matrix which was then subjected to multidimensional scaling analysis and plotted in two dimensions (principal component axes) [31].

The association between SNP genotype and nasal carriage of *S. aureus* (binary trait) was tested in GenABEL using an allelic association test with 1 df. We included farm as a covariate in the model. To adjust for multiple testing with a high number of SNPs, we derived the empirical distribution of the chi-square statistic after 10,000 permutations of the whole dataset. Genome-wide significance was set to empirical *p*-values < 0.05. We used genomic control [33] to adjust for any inflation of the test statistic. Calculations of linkage disequilibrium (LD) and visualization of LD were performed in Haploview version 4.2 [34]. Since the annotation of the pig genome sequence is incomplete, we also interrogated the human orthologue of the candidate region (HSA: 17q12) using builds Sscrofa10.2 and GRCh38 accessed through the Ensembl genome browser (www.ensembl.org).

The association between total number of *spa* types colonizing over the three-week period (1, 2, or 3) and SNP MARC0099960 genotype (GG, GA, AA) was tested using Fisher’s Exact Test for Count Data. We tested if colonization by each clonal complex (CC9, CC30, and CC398) was non-random among SNP MARC0099960 genotypes using Fisher’s Exact Test for Count Data. In addition to the genotype model, we also tested a dominance/recessive model (GG vs. GA and AA; GG and GA vs. AA).

## Results

**Fig. 1:**
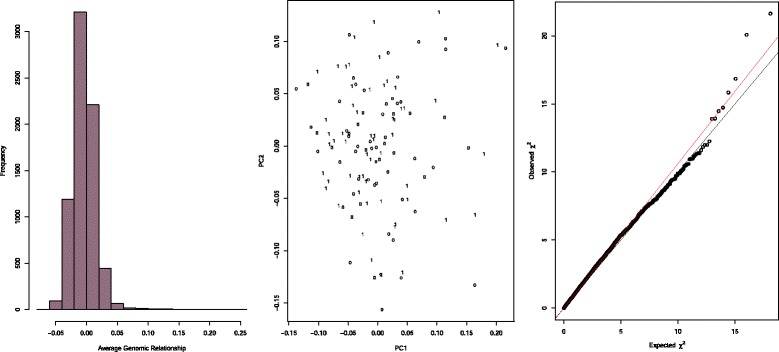
From left to right: **a** Distribution of genomic relationships between pairs of pigs (kinship matrix). **b** Multidimensional scaling plot (*n* = 121). 0, non-carriers; 1, persistent carriers; PC, principal component. **c** Quantile-quantile plot. Black line, the expected distribution of association test statistics under the null hypothesis of no association is plotted against observed values. Any deviation from the X-Y line suggests a consistent difference between persistent carriers and non-carriers e.g., due to genetic confounding. At the extreme of the distribution, the observed chi-square values are higher than expected by chance which indicates true association. Red line, fitted slope

The 23,919 SNPs which passed quality testing were used to compute a genomic kinship matrix for all pairs of pigs (Fig. 1a). Inspection of the matrix suggested that the pigs in our sample were only distantly related. We next applied multidimensional scaling to a distance matrix calculated from the genomic kinship matrix (Fig. 1b). The plot did not suggest any stratification of data with respect to *S. aureus* carriage (persistent carriers vs. non-carriers) or farm (data not shown). We constructed a quantile-quantile plot (Fig. 1c) and calculated the genomic inflation factor (λ_GC_ = 1.06) which also confirmed that genetic confounding was not an issue in our dataset.

**Fig. 2:**
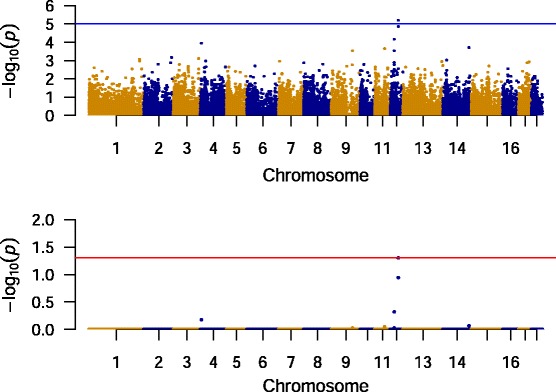
Manhattan plots for GWAS of nasal *Staphylococcus aureus* carriage in Danish crossbred pigs. The analysis included 65 non-carriers and 56 persistent carriers which were genotyped at 23,919 autosomal SNPs. *p*-values were corrected using lambda statistic to account for genetic confounding. Upper figure shows raw *p*-values; a suggestive significance threshold (*p* = 1 ×10^−5^ is indicated with a horizontal line. Lower figure shows permuted dataset after 10,000 permutations; the horizontal line shows the genome-wide significance threshold (*p* = 0.05)

A GWAS was performed on the final dataset (Fig. 2). One locus (SNP MARC0099960) on porcine chromosome 12 demonstrated association with carriage of *S. aureus*. The effect was genome-wide significant after permutation testing (*p* < 0.05; Table 1; Fig. 2). A neighboring SNP, ALGA0104951, in high LD with MARC0099960 (r^2^ = 0.806), did not reach genome-wide significance but was borderline significant after permutation testing (Table 1). These two SNPs are both located in an intergenic region.

**Table 1:** Statistics for two lead single nucleotide polymorphisms (SNPs) associated with nasal carriage of *Staphylococcus aureus* in Danish crossbred pigs (56 persistent carriers and 65 non-carriers)

SNP ID	Chromosome	Position (bp)	A1/A2	MAF (persistent carriers)	MAF (non-carriers)	*P* ^a^	*P* ^b^
MARC0099960	12	43,145,785	G/A	0.63	0.32	6.37×10^−6^	0.0497
ALGA0104951	12	43,380,247	G/T	0.61	0.33	1.38×10^−5^	0.1135

*MAF* minor allele frequency; *A1* minor allele; *A2* major allele

^a^Allelic association statistic adjusted for genomic control (*Pc1df*)

^b^Permutation test statistic after 10,000 permutations adjusted for genomic control

**Fig. 3:**
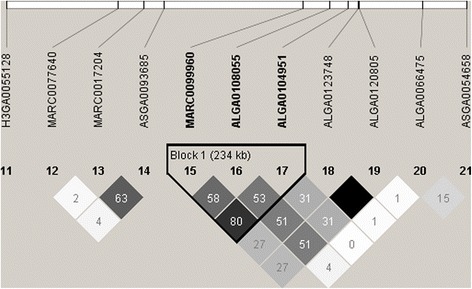
Linkage disequilibrium (LD) plot showing r^2^ x 100-values (correlation coefficient) with standard Haploview color scheme (ranging from white when r^2^ = 0 to black when r^2^ = 1). Block 1 indicates a region of 234 kb showing LD with SNP MARC0099960. The proposed candidate region is flanked by SNP markers ASGA0093685 and ALGA0123748, both showing no or week LD to block 1

To define the haplotype structure within the region, LD blocks were analyzed using Haploview. The measures of pairwise LD are shown in Fig. 3 where Block 1 indicates a region of 234 kb showing LD with SNP MARC0099960. The proposed candidate region is flanked by SNP markers ASGA0093685 and ALGA0123748, both showing no or weak LD with Block 1. Thus, the two SNPs (MARC0099960, ALGA0104951) delineate a haplotype block and since LD to flanking markers is weak we conservatively use these flanking markers as coordinates and delineate our QTL to SSC12: 42,422,021–43,436,573. With the limitations of the annotation of the porcine genome assembly, this QTL encompasses four annotated genes encoding chemokines (*CCL1*, *CCL2*, *CCL8*, *CCL11*).

The following 11 *spa* types were observed among the 126 *S. aureus* isolates from persistent carriers (frequency in brackets): t011 (11 %), t034 (76 %), t337 (5 %), t1333 (16 %), t1334 (8 %), t1580 (3 %), t2315 (16 %), t2370 (3 %), t2462 (11 %), t3131 (14 %), and t5817 (5 %). Thirteen pigs (35 %) were found to carry the same strain throughout the study, while the remaining 24 pigs carried either two (59 %) or three (5 %) different strains (Additional file 1: Table S1). The identified *spa* types have previously been associated with CC9 (t337, t1334, t2315, t2462, t3131) [35–37], CC30 (t1333) [38] and CC398 (t011, t034, t1580, t2370) [35, 39]. We did not find any reported clonal complex association for *spa* type t5817, which belonged to CC9 according to BURP cluster analysis. The association between SNP MARC0099960 and host colonization was independent of *S. aureus* genotype.

## Discussion

To our knowledge the work presented here is the first attempt to decipher the host genetic factors involved in nasal carriage of *S. aureus* in pigs. The GWAS demonstrated a significant association between a SNP marker located in a non-coding region (SNP MARC0099960) and nasal carriage of *S. aureus*, while the association of a nearby polymorphism in high LD with SNP MARC0099960 (SNP ALGA0104951) was borderline significant. Regardless, the results presented here are preliminary and the association should be replicated in other pigs to confirm the findings.

The frequency of the G allele at the SNP MARC0099960 locus was twice as high in carrier pigs compared to non-carriers of *S. aureus* (Table 1). The majority (31 out of 36) of the 121 genotyped pigs that were homozygous for the A allele were non-carriers of *S. aureus* while the majority (20 out of 28) that were homozygous for the G allele were persistent carriers, suggesting that the G allele is associated with susceptibility to nasal carriage of *S. aureus*. A total of 31 and 26 pigs were heterozygotes in the group of persistent- and non-carriers, respectively. Since both SNPs are located in a non-coding part of the genome, our results indicate that the haplotype tagged by these two SNPs contains one or several genes with an effect on *S. aureus* nasal carriage.

We used the Ensembl genome browser to interrogate our region of interest (build Sscrofa10.2) and the human orthologue of the region of interest (build GRCh38). Inspection of our ~1 Mb QTL region revealed that it encompasses a cluster of four chemokine genes (*CCL1*, *CCL2*, *CCL8*, and *CCL11*). A causative variant may be a SNP located in an exon of a protein-coding gene (changing the amino acid sequence of the protein), a regulatory part of a gene (altering the expression level), or a copy-number variant of a gene [40, 41]. Chemokines are expressed by a variety of cells to help direct immune cells of the innate and adaptive branch of the immune system to the site of foreign antigen [42]. There is evidence suggesting some of our candidate chemokines may be invoked following bacterial colonization; *S. aureus* antigens have been shown to stimulate expression of CCL1 by dendritic cells [43] while another study demonstrated human alveolar epithelial cells produced CCL2 following stimulation by LPS, a component of the gram-negative cell wall [44]. CCL2 has chemotactic properties for monocytes [45]; indeed, recruitment of macrophages required expression of the CCL2-binding chemokine receptor 2 in a mouse model of *Steptococcus pneumoniae* colonization [46]. CCL1 and CCL11 had direct antimicrobial activity against *S. aureus* while CCL2 and CCL8 did not have any effect on this pathogen [47].

The host factors underlying the differences in *S. aureus* carriage are not yet fully understood [6]. Studies in a murine model have suggested *S. aureus* clearance is T-cell mediated and happens via an IL-17A-dependent recruitment of neutrophils [48]. While the adaptive immune response was found to be important, these authors were not able to demonstrate that B-cells were crucial. In agreement with these findings there is evidence showing that immunity to pneumococcal colonization is antibody independent but does require CD4^+^ T cells [49].

Research in humans and murine models using infection with *S. aureus* as phenotypic trait have suggested different positional candidate genes, e.g., *SEH1L*, *TNFAIP8*, *KLK*, and *CDON* [16, 17, 50, 51]. However, none of these genes are situated in or close to our QTL region. Genetic studies in human populations are challenged by a considerable genetic heterogeneity which may explain why previous efforts have shown a non-significant heritability of nasal carriage of *S. aureus* [9, 10]. In contrast, pigs may be used as a convenient model since they are much less heterogeneous and smaller sample sizes are needed to detect genetic variants associated with complex traits [52]. The pig model may be used to further explore *S. aureus* colonization mechanisms in humans; e.g., by taking advantage of the possibility to control various factors under experimental settings (e.g., housing conditions, known inoculation doses, known pedigrees, etc.).

GWAS for host susceptibility to infectious pathogens should take the genome of the microorganism, i.e., strain information, into account [53]. This notion is particularly relevant to *S. aureus* colonization, which is the result of a complex interplay between host and bacterial factors [54–56]. All *S. aureus spa* types identified in the present study have previously been isolated from pigs [35, 36, 38, 39] except t3131 (CC9) which has only been reported in cattle [37]. CC398-associated *spa* types were isolated from most (33/37) persistent carriers, suggesting that CC398 is the most prevalent *S. aureus* lineage in Danish crossbreed pigs. Even though the study was not designed to study coexistence of different lineages in the nasal cavity of pigs (i.e., only one or two isolates were *spa* typed from each sample), our results show that persistent carriers can be colonized by several lineages during a period of three weeks and by more than one strain at the same time.

The association between SNP MARC0099960 and host colonization was independent of *S. aureus* genotype. This is valuable information considering that *S. aureus* is a highly clonal microorganism and one clonal lineage (CC398) accounts for most livestock-associated MRSA infections in Europe [20, 21]. Identification of genetic markers associated with nasal carriage of *S. aureus* may be used in breeding to select animals with reduced susceptibility to colonization by this organism. Such a breeding program may serve as an unexplored option to prevent spread of livestock-associated MRSA to humans. A similar approach was used to detect the locus responsible for enterotoxigenic *E. coli* diarrhea in piglets, and a genetic marker test which distinguishes between susceptible and resistant animals has been developed [57]. The findings presented here may also improve our understanding of the host mechanisms underlying *S. aureus* colonization in both pigs and humans. The new locus detected in the present study provides a basis for further exploration by validation and functional testing of the markers and candidate genes.

## Conclusion

We have identified significant association between a SNP marker located in a non-coding region (SNP MARC0099960) and nasal carriage of *S. aureus*. The QTL region encompasses a cluster of four chemokine genes (*CCL1, CCL2, CCL8*, and *CCL11*) which are potential candidate genes for nasal carrige. Our results suggest it may be possible to select pigs genetically resistant to *S. aureus* nasal colonization as a tool to control transmission of livestock-associated MRSA to humans.

## Additional file

Additional file 1: Table S1. Pig and *spa* type distribution across farms. (PDF 16 kb)

## Abbreviations

*S. aureus*: *Staphylococcus aureus*
CC: Clonal complex
MRSA: Methicillin-resistant *Staphylococcus aureus*
