# Supplementary material for: Genome-wide association study reveals a locus for nasal carriage of Staphylococcus aureus in Danish crossbred pigs
Source: BMC Vet Res. 2015 Nov 26;11:290. doi: 10.1186/s12917-015-0599-y (PMC4662016; doi:10.1186/s12917-015-0599-y)
Supplement: Additional file 1: Table S1. — Pig and spa type distribution across farms. (PDF 16 kb) [file 12917_2015_599_MOESM1_ESM.pdf]

TABLE S1 Pig and *spa* type distribution across farms.

Distribution across farms of Danish crossbred pigs included in genome-wide association study. The pigs were classified as non-carriers or persistent carriers of *Staphylococcus aureus.* Also shown is the *spa* type distribution among 37 persistent carriers.

.

Farm No.	Non-carriers	Persistent carriers (numbers spa-typed)	Number of persistent carriers colonized by one *spa* type	Number of persistent carriers colonized by two *spa* types	Number of persistent carriers colonized by three *spa* types
2	5	4 (3)	3 (t011)	0	0
3	0	6 (5)	0	5 (t034, t1334, t2462)	0
4	4	0	NA	NA	NA
5	0	5 (5)	2 (t034, t1333)	3 (t034, t1333)	0
6	5	2 (2)	0	2 (t034, t1333)	0
7	10	0	NA	NA	NA
8	12	1 (0)	NA	NA	NA
10	0	12 (12)	7 (t034)	5 (t034, t3131)	0
11	0	2 (1)	0	0	1 (t011, t1580, t5817)
12	1	7 (6)	1 (t2315)	4 (t034, t2315, t5817)	1 (t2315, t2370, t5817)
13	16	1 (1)	0	1 (t034, t1334)	0
16	5	2 (2)	0	2 (t034, t337)	0
17	2	5 (0)	NA	NA	NA
18	7	6 (0)	NA	NA	NA
20	5	3 (0)	NA	NA	NA
Total	65	56 (37)	13	22	2

NA, not applicable.
